# Supplementary material for: Antimalarial Peptide and Polyketide Natural Products from the Fijian Marine Cyanobacterium Moorea producens
Source: Mar Drugs. 2020 Mar 18;18(3):167. doi: 10.3390/md18030167 (PMC7142784; doi:10.3390/md18030167)
Supplement: Supplementary file 1 [file marinedrugs-18-00167-s001.pdf]

Supplementary Materials:

Antimalarial Peptide and Polyketide Natural Products from the Fijian Marine Cyanobacterium *Moorea producens*

Anne Marie Sweeney-Jones [1], Kerstin Gagaring [2], Jenya Antonova-Koch [2], Hongyi Zhou [3], Nazia Mojib [3], Katy Soapi [4], Jeffrey Skolnick [3,5], Case W. McNamara [2], and Julia Kubanek [1,3,5,6,*]

[1] School of Chemistry and Biochemistry, Georgia Institute of Technology, Atlanta, GA 30332, United States; amsj3@gatech.edu

[2] Calibr, a division of The Scripps Research Institute, La Jolla, CA 92037, United States; kerstin.gagaring@gmail.com (K.G.); jantonovakoch@scripps.edu (J.A.K.); cmcnamara@scripps.edu (C.W.M.)

[3] School of Biological Sciences, Georgia Institute of Technology, Atlanta, GA 30332, United States; hongyi.zhou@biology.gatech.edu (H.Z.); nazia.mojib@biosci.gatech.edu (N.M); skolnick@gatech.edu (J.S.)

[4] Institute of Applied Sciences, University of the South Pacific, Suva, Fiji; katy.soapi@usp.ac.fj

[5] Parker H. Petit Institute for Bioengineering and Bioscience, Georgia Institute of Technology, Atlanta, GA 30332, United States

[6] Center for Microbial Dynamics and Infection, Georgia Institute of Technology, Atlanta, GA 30332, United States

* Correspondence: julia.kubanek@biosci.gatech.edu; Tel.: +1-404-894-8424

Table of Contents

Figure S1. Evolutionary relationship of *Moorea producens* G-0362 with closely related cyanobacterial species 3

Figure S2. HRESIMS of kakeromamide B (**1**) 4

Figure S3. ^{1}H NMR spectrum of kakeromamide B (**1**) 5

Figure S4. COSY spectrum of kakeromamide B (**1**) 6

Figure S5. HSQC NMR spectrum of kakeromamide B (**1**). 7

Figure S6. HMBC NMR spectrum of kakeromamide B (**1**). 8

Figure S7. HRESIMS of ulongamide A (**2**). 9

Figure S8. ^{1}H NMR spectrum of ulongamide A (**2**) 10

Figure S9. COSY spectrum of ulongamide A (**2**) 11

Table S1. Comparison of experimental and literature chemical shifts of ulongamide A (**2**) 12

Figure S10. HRESIMS of lyngbyabellin A (**3**) 13

Figure S11. ^{1}H NMR spectrum of lyngbyabellin A (**3**) 14

Figure S12. COSY NMR spectrum of lyngbyabellin A (**3**) 15

Table S2. Comparison of experimental and literature chemical shifts of lyngbyabellin A (**3**). 16

Figure S13. HRESIMS of 18*E*-lyngbyaloside C (**4**) 17

Figure S14. ^{1}H NMR spectrum for 18*E*-lyngbyaloside C (**4**) 18

Figure S15. COSY NMR spectrum for 18*E*-lyngbyaloside C (**4**) 19

Table S3. Comparison of experimental and literature chemical shifts of *18E*-lyngbyaloside C (**4**)...............20
Figure S16. HRESIMS of lyngbyaloside (**5**)...22
Figure S17. ^{1}H NMR spectrum for lyngbyaloside (**5**) 23
Figure S18. COSY NMR spectrum for lyngbyaloside (**5**) 24
Table S4. Comparison of experimental and literature chemical shifts of lyngbyaloside (5). 25
Figure S19. HRESIMS of lyngbyabellin-like 1 (**LYN1**)..27
Figure S20. ^{1}H NMR spectrum of lyngbyabellin-like 1 (**LYN1**)...28
Figure S21. HRESIMS of lyngbyabellin-like 2 (**LYN2**)..29
Figure S22. ^{1}H NMR spectrum of lyngbyabellin-like 2 (**LYN2**)...30
Figure S23. Effect of latrunculin A and kakeromamide B (**1**) on actin polymerization 31

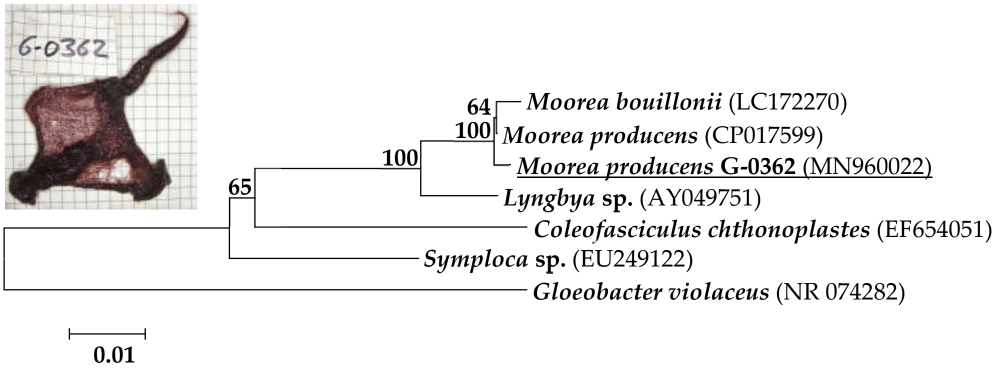

Figure S1. Evolutionary relationship of *Moorea producens* G-0362 with closely related cyanobacterial species from the Family Oscillatoriaceae inferred by the 16S rRNA sequences using the Maximum Likelihood method based on the Kimura 2-parameter model in MEGA X. The percentage of replicate trees in which the associated species clustered together in the bootstrap test (1000 iterations) is shown next to the branches. The accession numbers of the 16S rRNA of respective cyanobacteria are mentioned in parentheses. The outgroup taxon is *Gloeobacter violaceus*. The 16S rRNA sequence from *Moorea producens* used in this study is underlined. The tree is drawn to scale, with branch lengths measured in the number of substitutions per site.

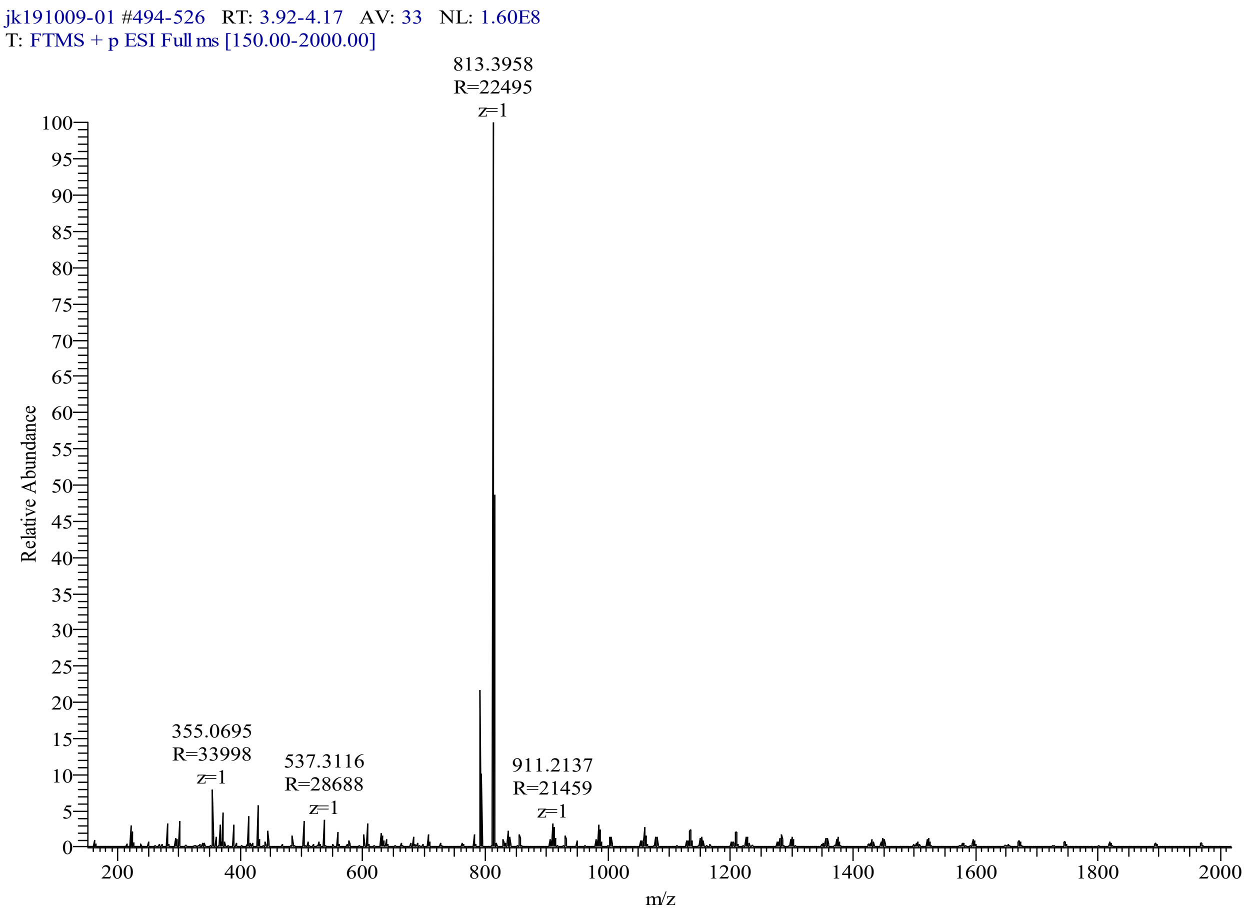

Figure S2. HRESIMS of kakeromamide B (**1**), *m*/*z* $[M+H]^+$ calculated for $C_{42}H_{59}N_6O_7S$ 791.4166, found 791.4150 (Δ = 2.0 ppm); $[M+Na]^+$ calculated for $C_{42}H_{58}N_6O_7SNa$ 813.3985, found 813.3958 (Δ = 3.3 ppm).

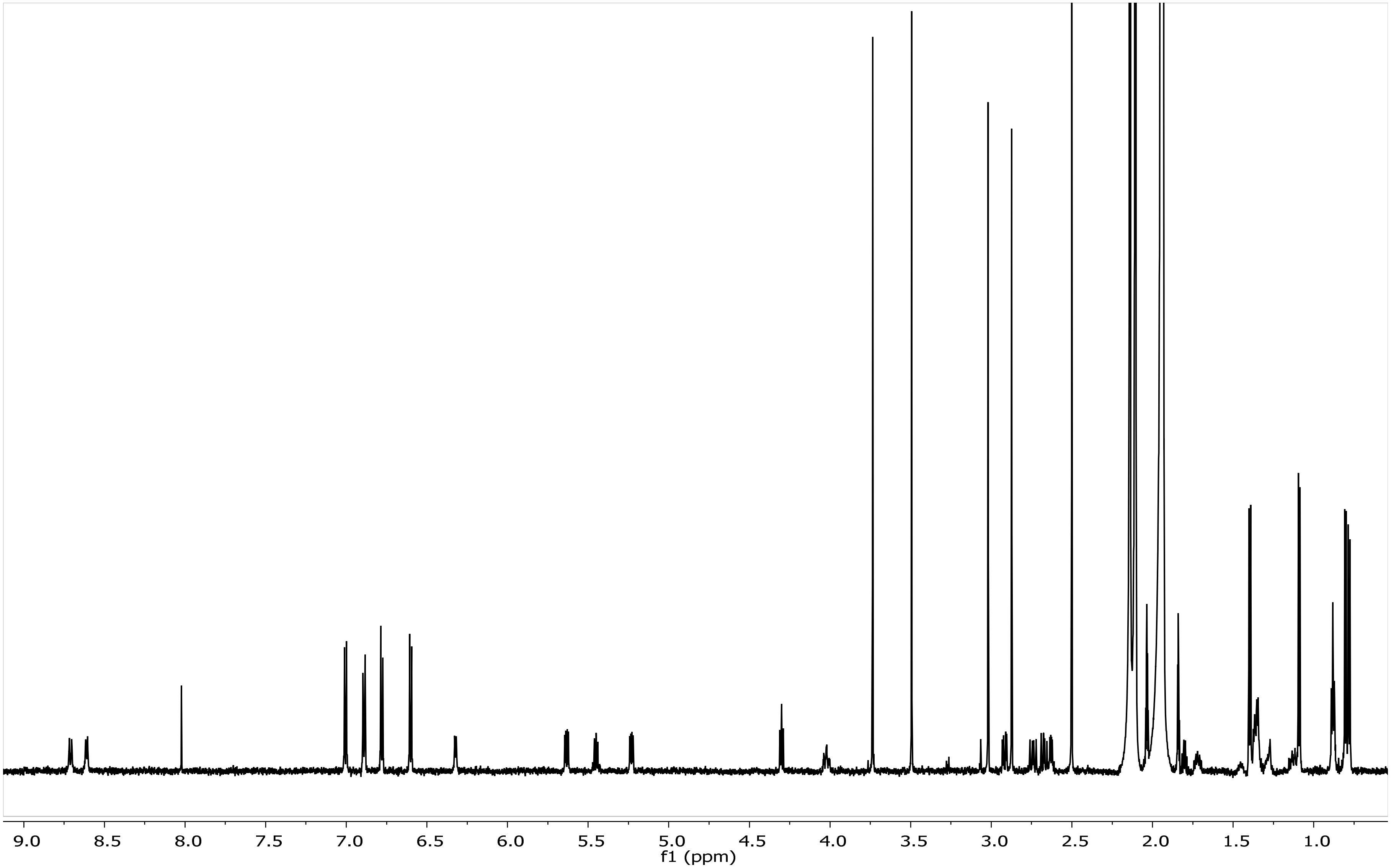

Figure S3. ^{1}H NMR spectrum of kakeromamide B (**1**) in CD_3CN (700 MHz).

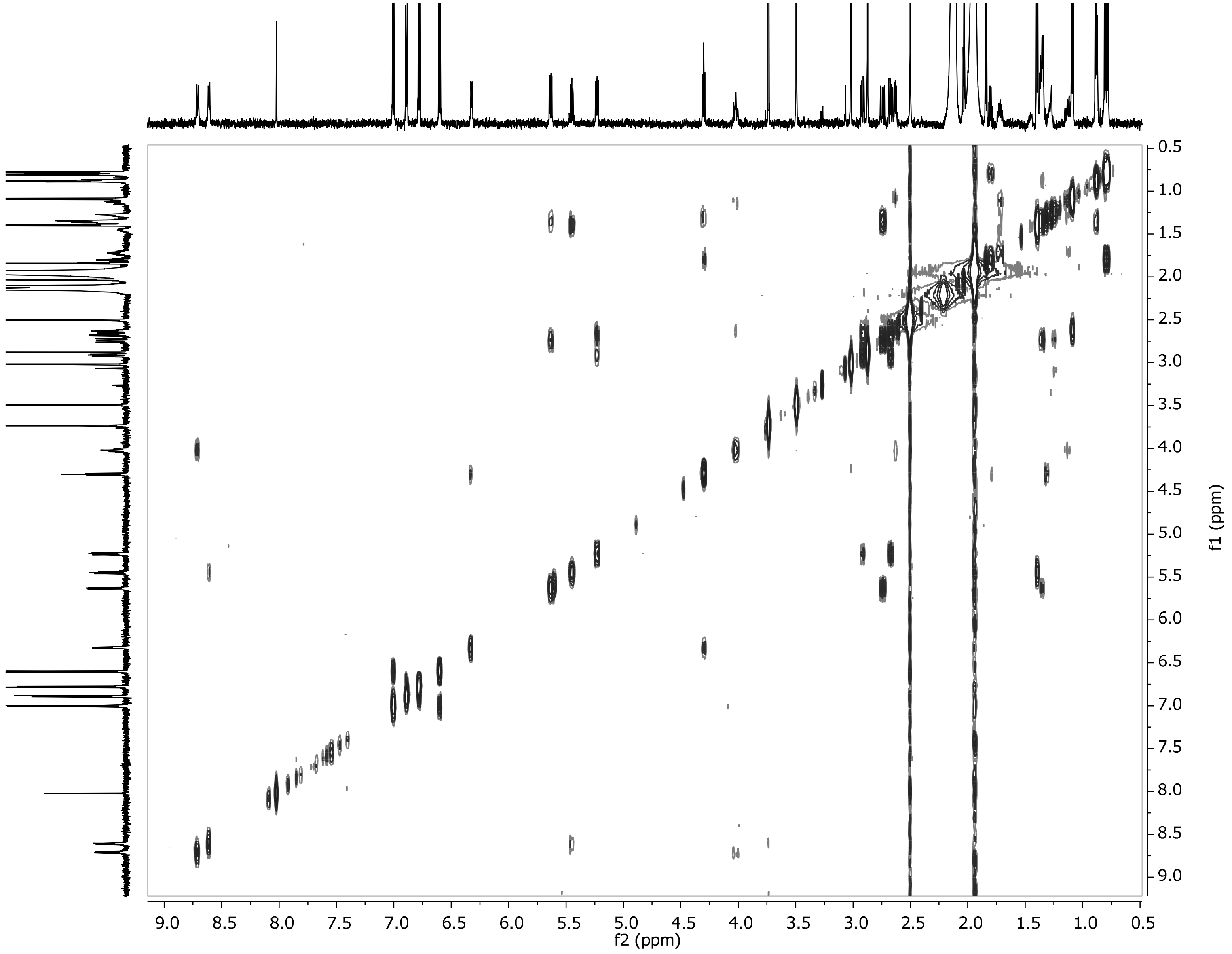

Figure S4. COSY spectrum of kakeromamide B (**1**) in CD_3CN (700 MHz).

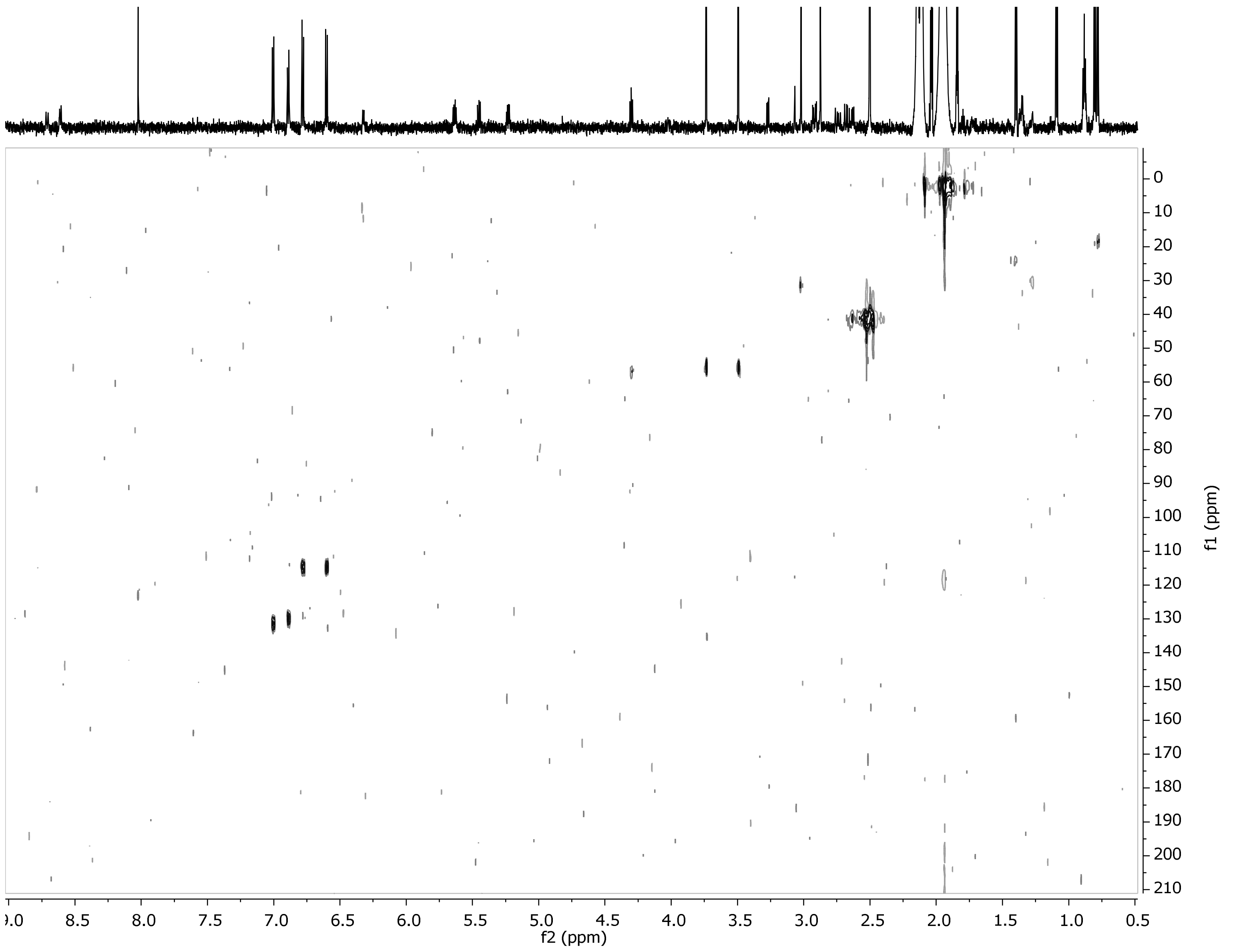

Figure S5. HSQC NMR spectrum of kakeromamide B (**1**) in CD_3CN (700 MHz).

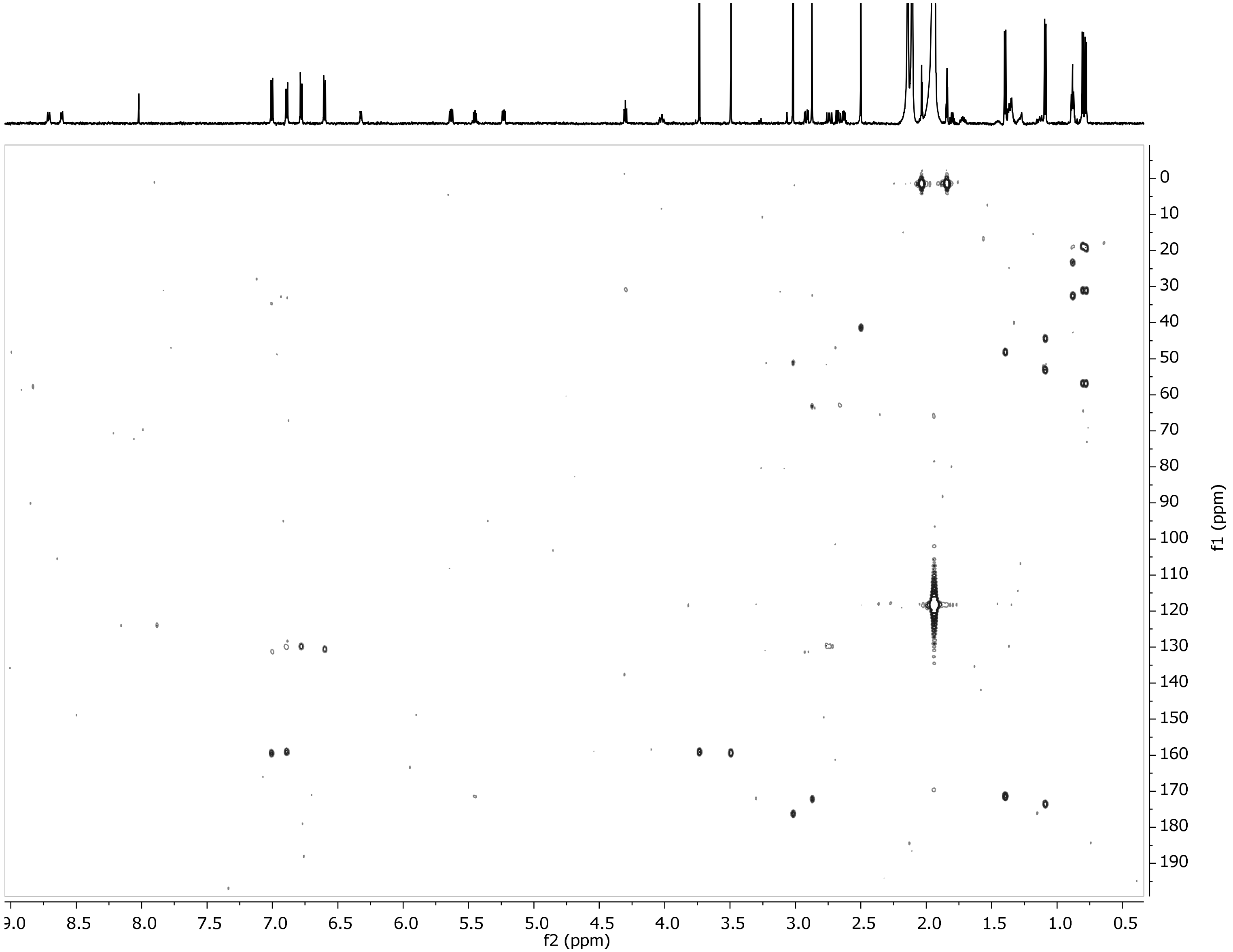

Figure S6. HMBC NMR spectrum of kakeromamide B (**1**) in CD_3CN (700 MHz).

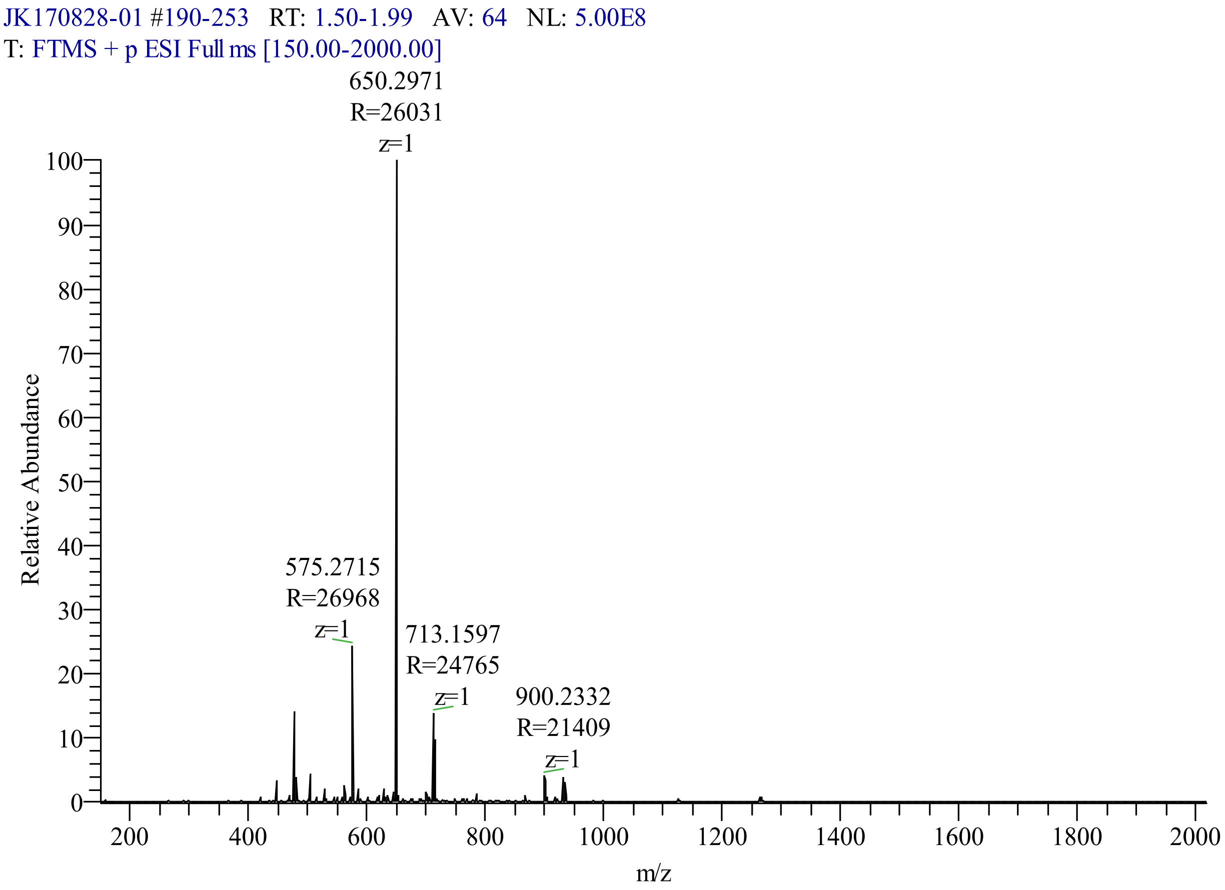

Figure S7. HRESIMS of ulongamide A (**2**), *m/z* [M + Na]+ calculated for $C_{32}H_{45}N_5O_6SNa$ 650.2983, found 650.2971 (Δ = 1.8 ppm).

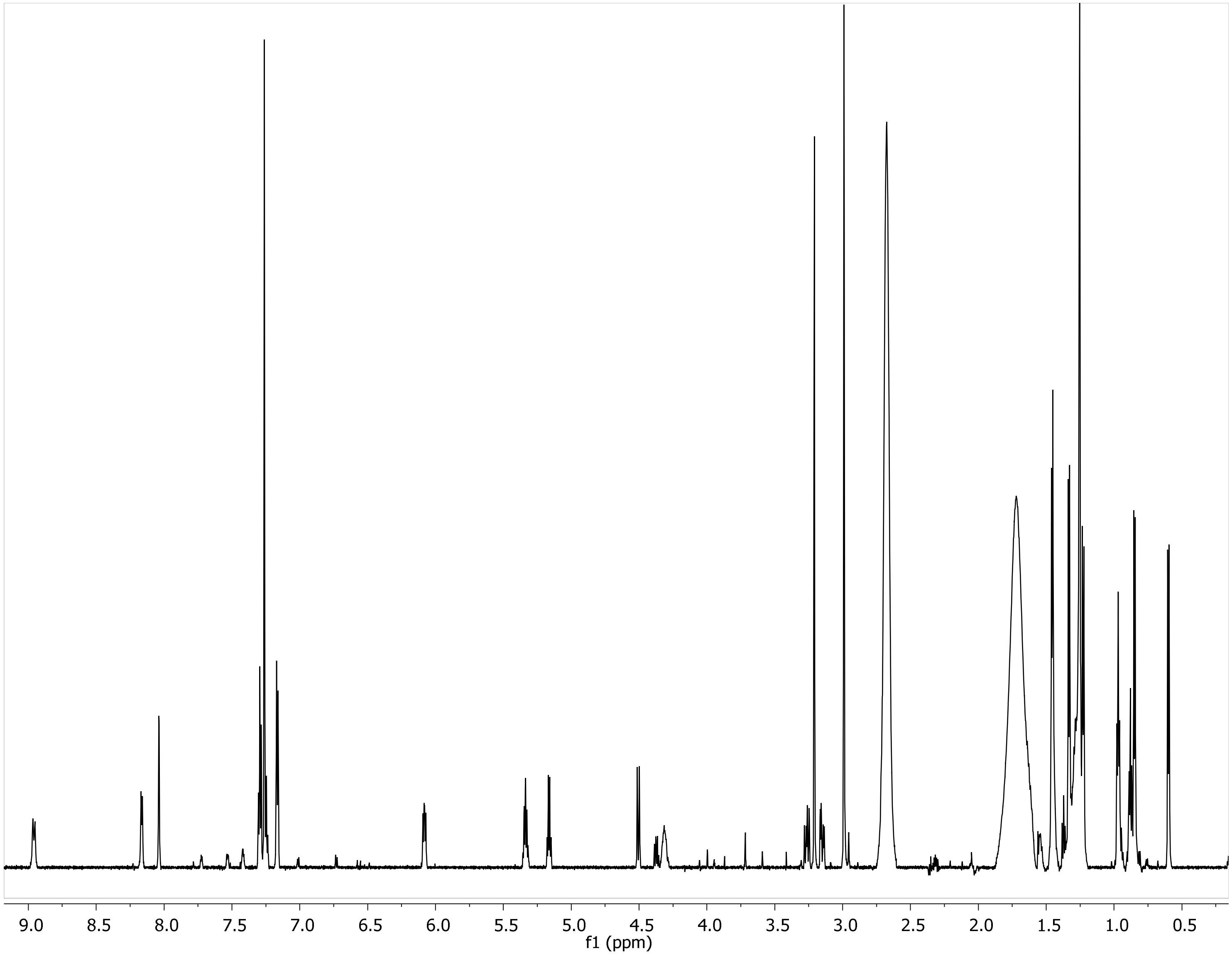

Figure S8. ^{1}H NMR spectrum of ulongamide A (**2**) in $CDCl_3$ (700 MHz).

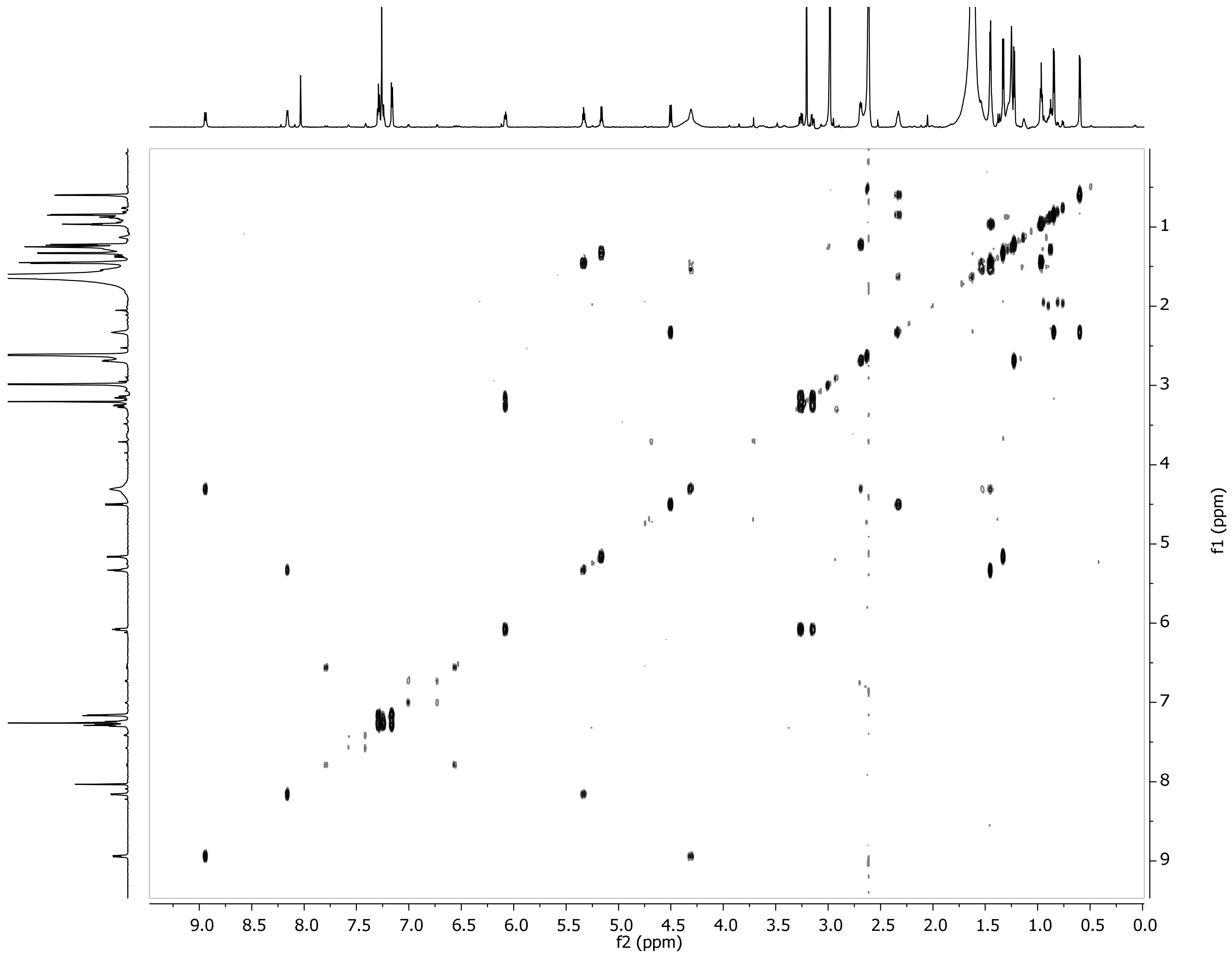

Figure S9. COSY spectrum of ulongamide A (**2**) in $CDCl_3$ (800 MHz).

Table S1. Comparison of ^{1}H NMR chemical shifts of ulongamide A (**2**) reported in literature [1] (500 MHz, $CDCl_3$) to experimental values of **2** (700 MHz, $CDCl_3$).

Unit	Literature Values		Experimental Values (2)	Δ (ppm)
	C/H no.	δ_H (*J* in Hz)	δ_H (*J* in Hz)	
**Amha**	1			
	2	2.68, qd (6.9, 2.8)	2.69, m	0.01
	3	4.31, m	4.31, m	0
	4a	1.43, m	1.43, m	0
	4b	1.54, m	1.55, m	0.01
	5	1.43, m	1.43, m	0
	6	0.97, t (7.0)	0.97, t (7.0)	0
	7	1.22, d (6.9)	1.22, d (6.9)	0
	NH	8.95, d (10.8)	8.94, d (10.6)	0.01
**Ala-thz-ca**	1			
	2			
	3	8.04, s	8.03, s	0.01
	4			
	5	5.33, quint (6.6)	5.33, quint (6.6)	0
	6	1.45, d (6.6)	1.45, d (6.7)	0
	NH	8.17, d (6.6)	8.16, d (6.7)	0.01
***N*-Me-Val**	1			
	2	4.50, d (10.8)	4.50, d (10.8)	0
	3	2.33, m	2.33, m	0
	4	0.59, d (7.0)	0.60, d (6.8)	0.01
	5	0.84, d (6.6)	0.85, d (6.4)	0.01
	N-CH_3	2.99, s	2.99, s	0
***N*-Me-Phe**	1			
	2	6.08, dd (9.4, 5.5)	6.08, dd (9.4, 5.6)	0
	3a	3.14, dd (−15.2, 5.5)	3.15, dd (15.2, 5.5)	0.01
	3b	3.26, dd (−15.2, 9.4)	3.26, dd (15.1, 9.4)	0
	4			
	5/9	7.16, d (7.2)	7.16, d (7.5)	0
	6/8	7.29, m	7.29, t (7.5)	0
	7	7.25, m	7.24, m	0.01
	N-CH_3	3.20, s	3.20, s	0
**lactic acid**	1			
	2	5.15, q (6.6)	5.16, q (6.7)	0.01
	3	1.33, d (6.6)	1.33, d (6.7)	0

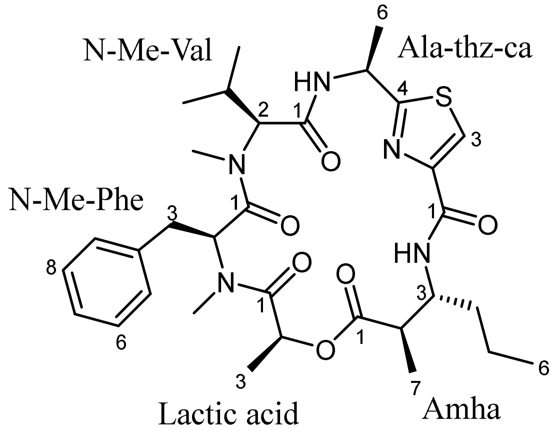

ulongamide A (**2**)

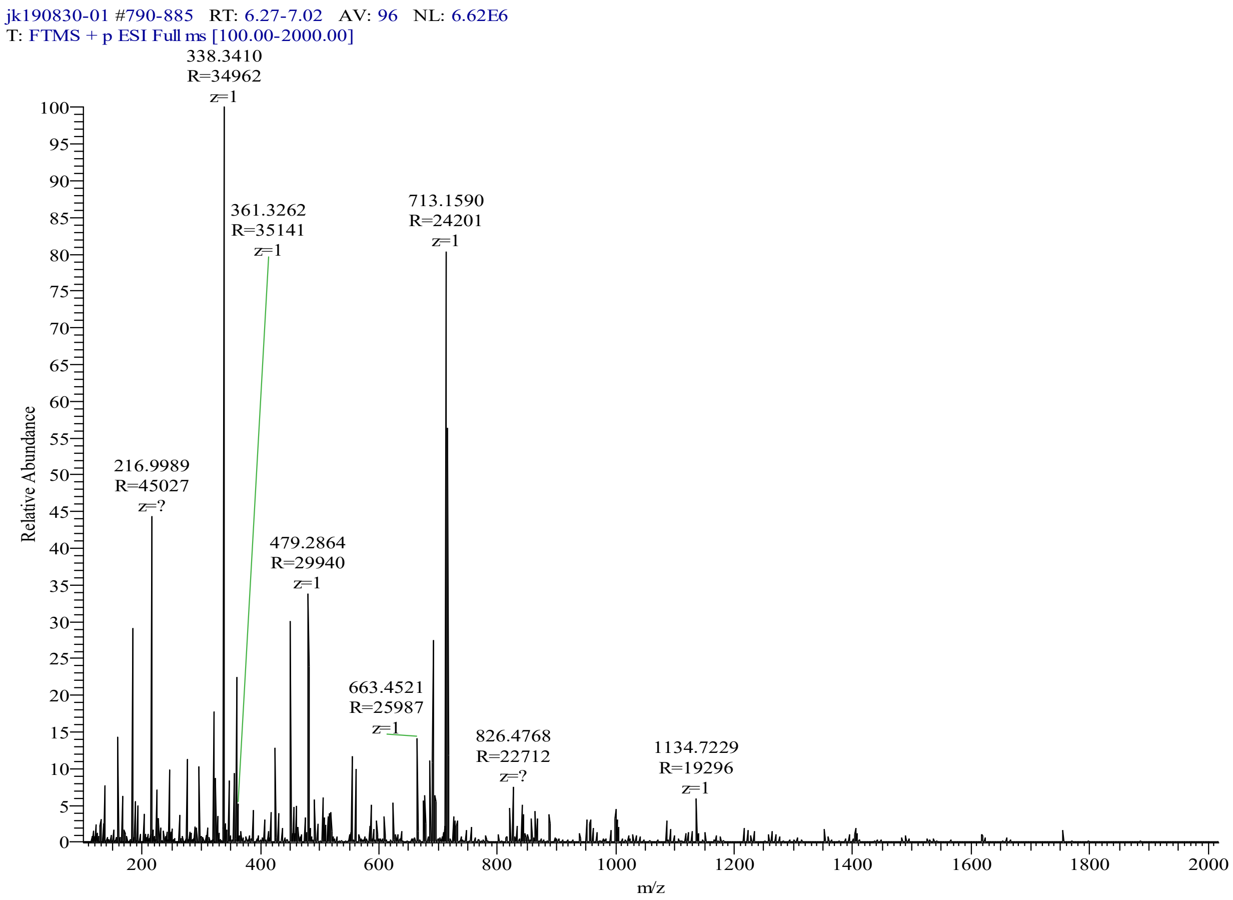

Figure S10. HRESIMS of lyngbyabellin A (**3**), *m/z* $[M + H]^+$ calculated for $C_{29}H_{41}Cl_2N_4O_7S_2$ 691.1788, found 691.1771 (Δ = 2.5 ppm); *m/z* $[M + Na]^+$ calculated for $C_{29}H_{40}Cl_2N_4O_7S_2Na$ 713.1608, found 713.1590 (Δ = 2.5 ppm).

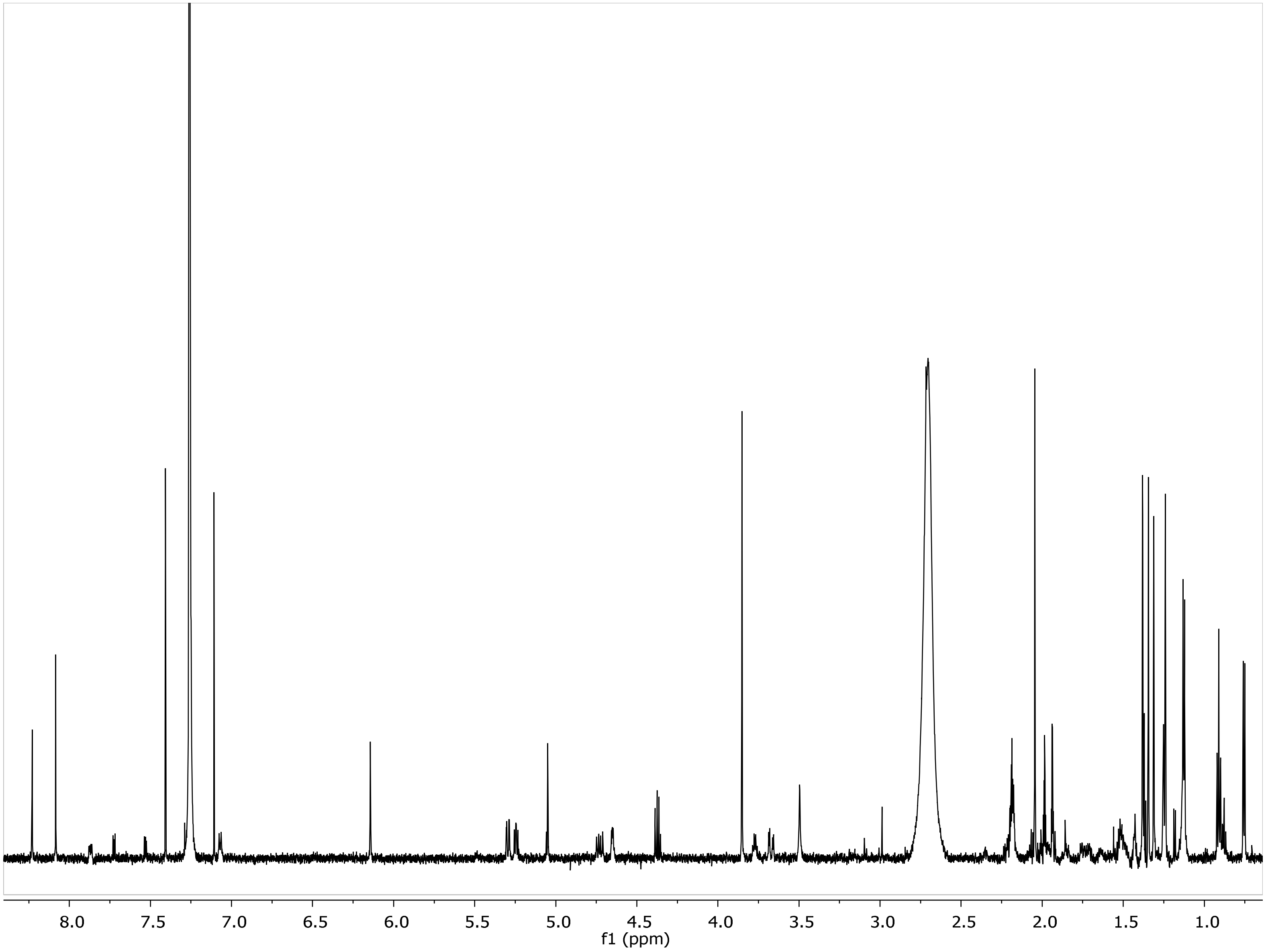

Figure S11. ^{1}H NMR spectrum of lyngbyabellin A (**3**) in $CDCl_3$ (700 MHz).

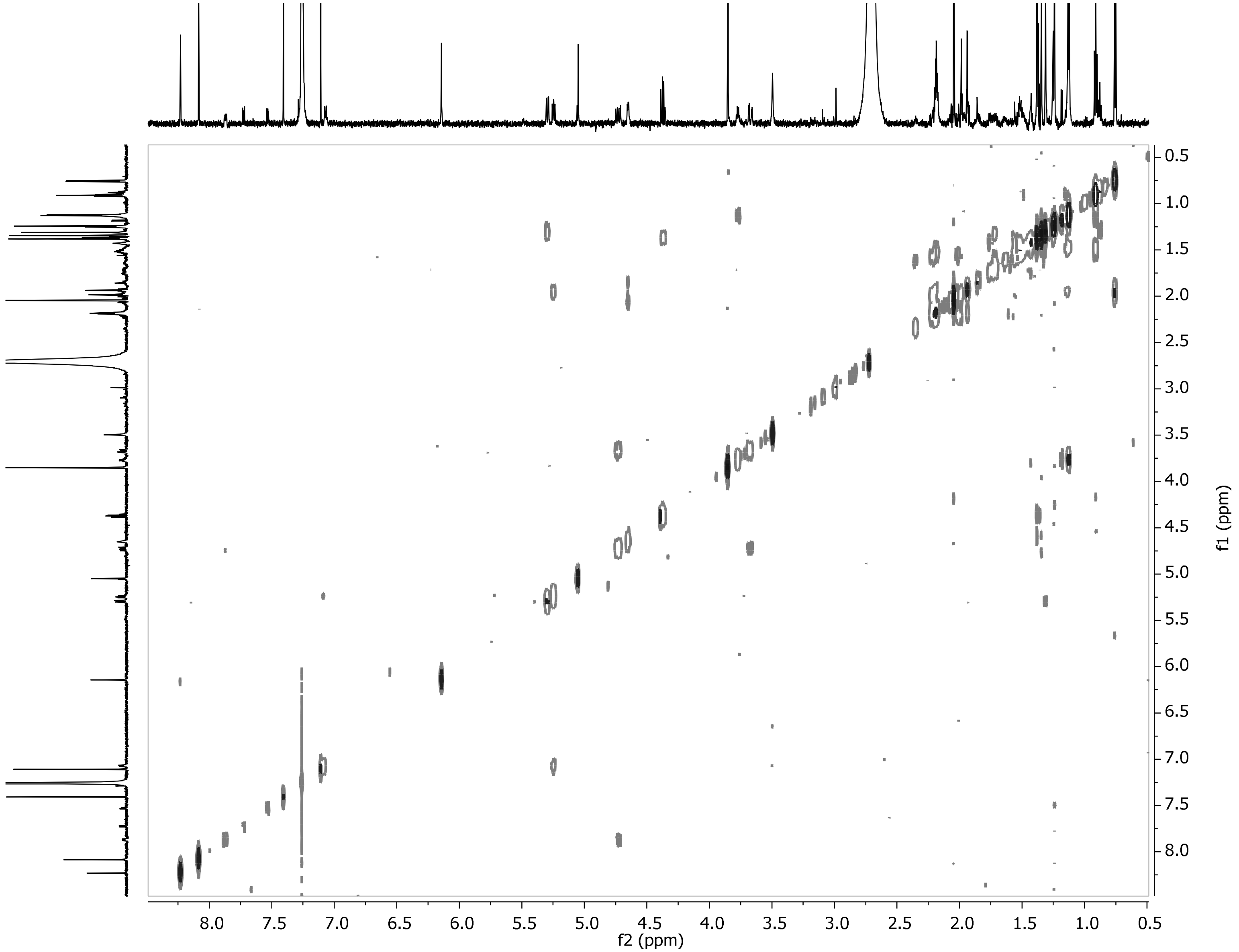

Figure S12. COSY NMR spectrum of lyngbyabellin A (**3**) in $CDCl_3$ (700 MHz).

Table S2. Comparison of ^{1}H NMR chemical shifts of lyngbyabellin A (**3**) reported in literature [2] (500 MHz, $CDCl_3$) to experimental values of **3** (700 MHz, $CDCl_3$).

C/H no.	Literature Values	Experimental Values (3)	Δ (ppm)
	δ_H (*J* in Hz)	δ_H (*J* in Hz)	
**1**			
**2**			
**3**	5.31, dd (10.6, 2.3)	5.30, dd (10.9, 2.3)	0.01
**4a**	1.33, m	1.31, m	0.02
**4b**	1.72, m	1.73, m	0.01
**5**	1.60, m	1.64, m	0.04
**6a**	2.00, m	1.99, p (2.4)	0.01
**6b**	2.22, ddd (−14.2, 10.8, 5.2)	2.22, m	0
**7**			
**8**	2.05, s	2.05, s	0
**9**	1.31, s	1.31, s	0
**10**	1.36, s	1.35, s	0.01
**11**			
**12**			
**13**	8.09, s	8.08, s	0.01
**14**			
**15**	5.24, dd (9.0, 6.8)	5.25, dd (8.6, 6.6)	0.01
**15-NH**	7.27, d (9.0)	7.07, d (9.0)	0.2
**16**	1.97, m	1.94, q (2.6)	0.03
**17a**	1.13, m	1.13, d (6.8)	0
**17b**	1.50, m	1.52, m	0.02
**18**	0.90, t (7.3)	0.91, t (7.4)	0.01
**19**	0.75, d (6.6)	0.76, d (6.8)	0.01
**20**			
**21a**	3.70, dd (−17.0, 4.3)	3.67, dd (17.1, 4.0)	0.03
**21b**	4.70, dd (−17.0, 9.2)	4.73, dd (17.1, 9.2)	0.03
**21-NH**	7.97, dd (9.2, 4.3)	7.87, m	0.1
**22**			
**23**			
**24**	8.23, d (0.8)	8.23, d (0.6)	0
**25**			
**26**	6.13, d (0.8)	6.14, d (0.6)	0.01
**27**			
**28**	1.24, s	1.24, s	0
**29**	1.38, s	1.38, s	0

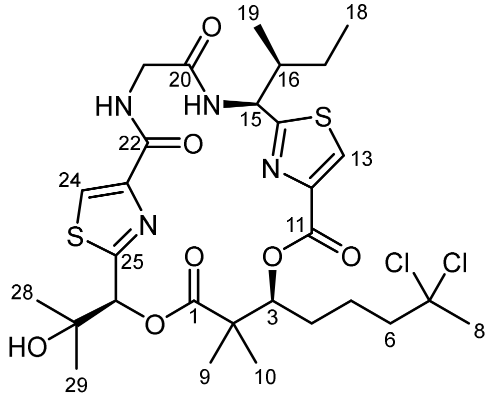

lyngbyabellin A (**3**)

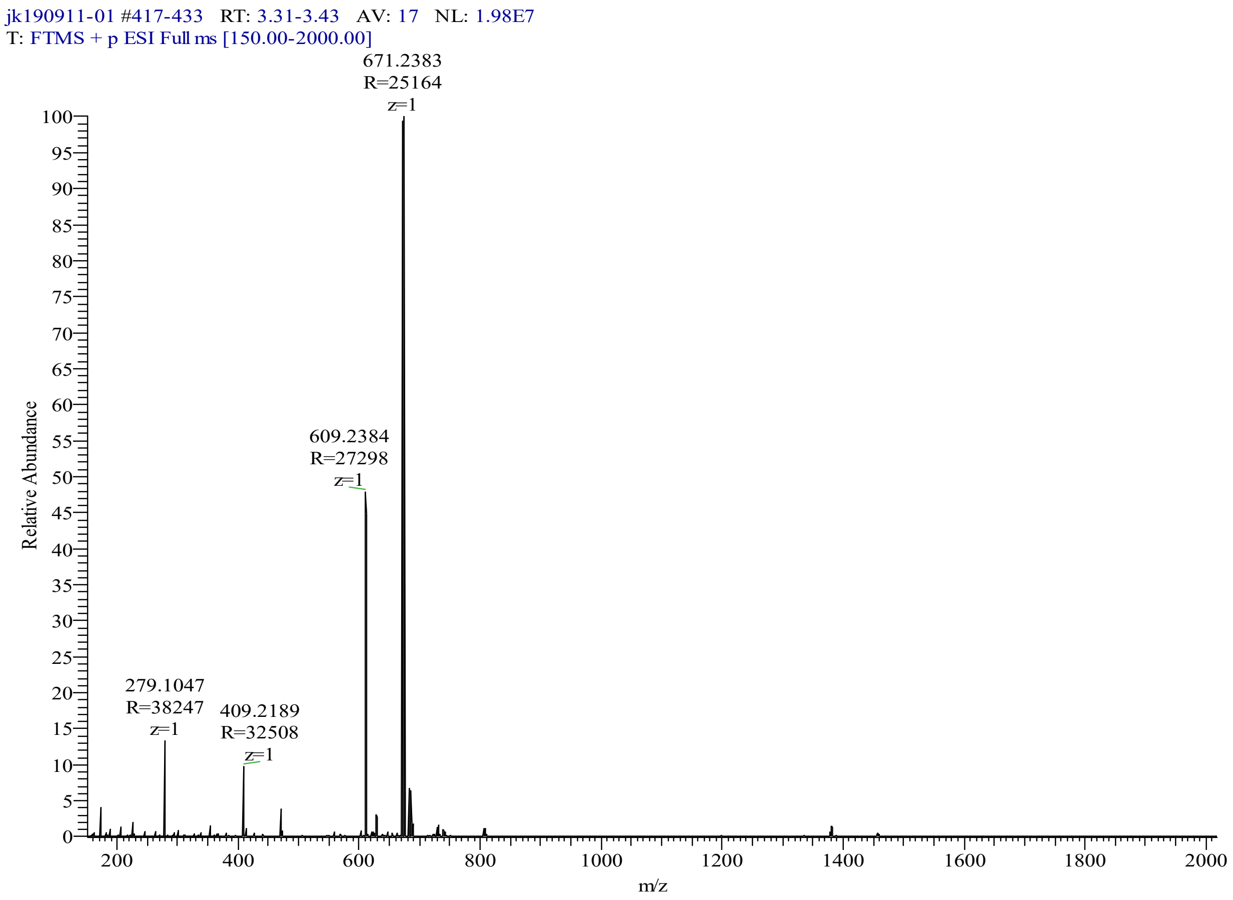

Figure S13. HRESIMS of 18*E*-lyngbyaloside C (**4**), *m*/*z* [M + Na]+ calculated for $C_{30}H_{49}BrO_{10}Na$ 671.2401, found 671.2383 (Δ = 2.7 ppm).

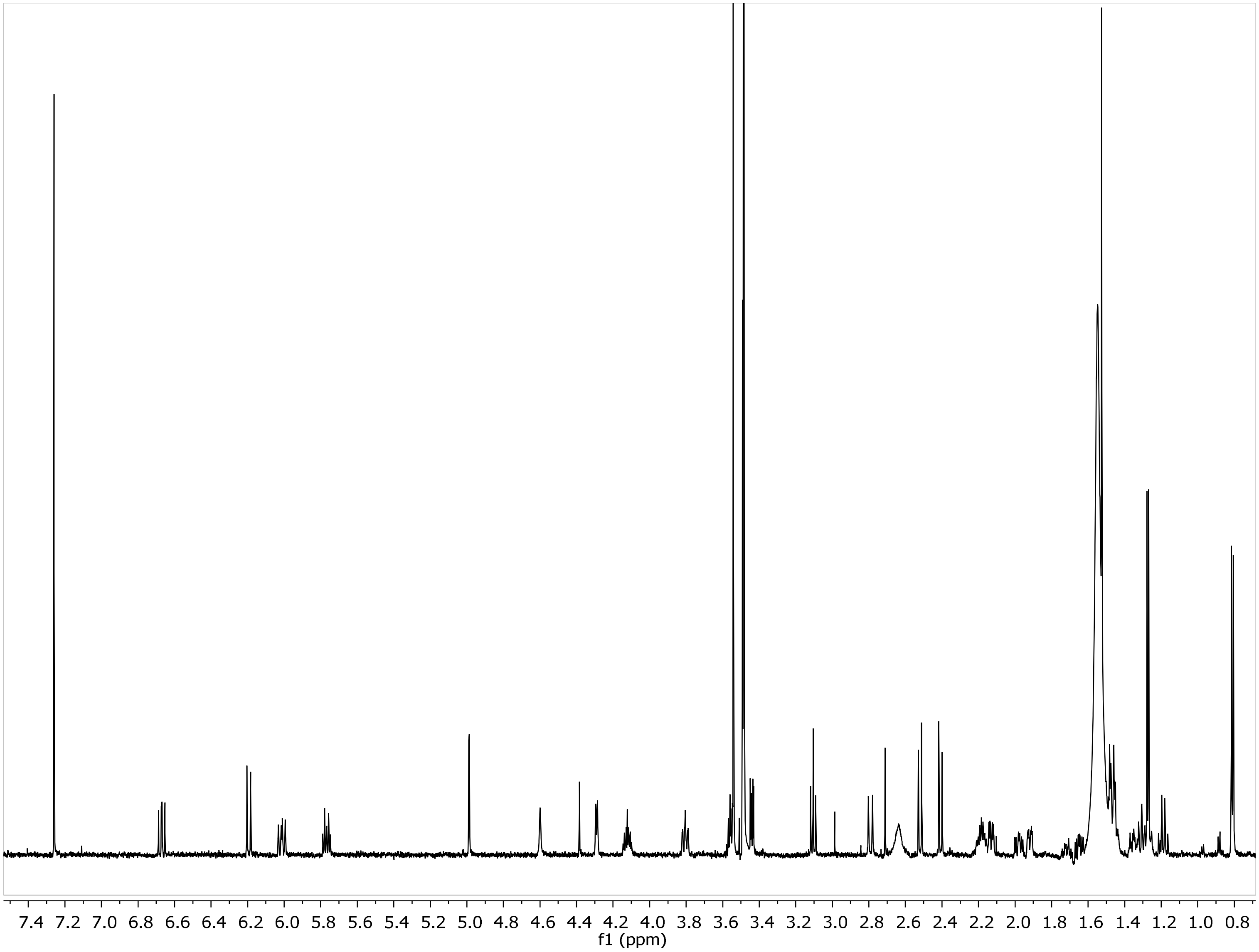

Figure S14. ^{1}H NMR spectrum for 18*E*-lyngbyaloside C (**4**) in $CDCl_3$ (700 MHz).

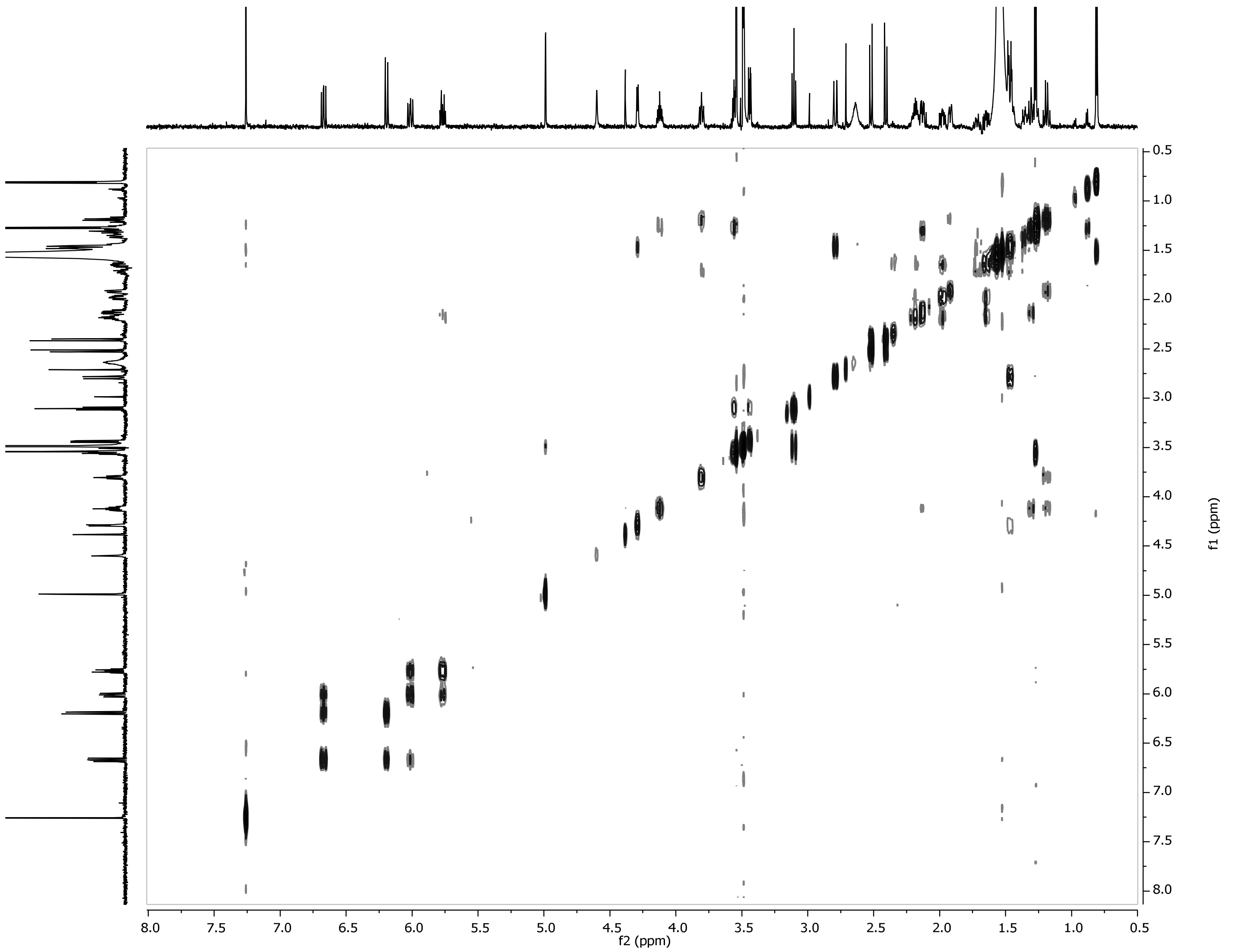

Figure S15. COSY NMR spectrum for 18*E*-lyngbyaloside C (**4**) in $CDCl_3$ (700 MHz).

Table S3. Comparison of ^{1}H NMR chemical shifts of *18E*-lyngbyaloside C (**4**) reported in literature [3,4] (600 MHz, $CDCl_3$) to experimental values of **4** (700 MHz, $CDCl_3$).

C/H no.	Literature Values	Experimental Values (4)	Δ (ppm)
	δ_H (*J* in Hz)	δ_H (*J* in Hz)	
**1**			
**2a**	2.49, d (−12.1)	2.52, d (12.2)	0.03
**2b**	2.38, d (−12.1)	2.41, d (12.2)	0.03
**3**			
**3-OH**	4.57, brs	4.60, s	0.03
**4a**	2.09, m	2.13, m	0.04
**4b**	1.28, m	1.26, m	0.02
**5**	4.10, m	4.12, m	0.02
**6a**	1.89, m	1.92, m	0.03
**6b**	1.15, m	1.19, m	0.04
**7**	3.79, t (10.1)	3.81, t (10.3)	0.02
**8a**	1.70, m	1.72, m	0.02
**8b**	1.45, m	1.46, m	0.01
**9a**	1.45, m	1.46, m	0.01
**9b**	1.32, m	1.30, m	0.02
**10**	1.48, m	1.48, m	0
**11**	4.26, m	4.29, m	0.03
**12a**	2.77, d (−15.6)	2.79, d (15.3)	0.02
**12b**	1.44, dd (−15.6, 5.3)	1.44, m	0
**13**			
**14a**	1.97, m	1.98, m	0.01
**14b**	1.63, m	1.65, m	0.02
**15a**	2.17, m	2.13, m	0.04
**15b**	2.18, m	2.18, m	0
**16**	5.76, dt (14.6, 5.9)	5.77, dt (14.3, 6.8)	0.01
**17**	5.99, dd (14.6, 10.6)	6.01, dd (15.2, 10.7)	0.02
**18**	6.63, dd (13.9, 10.6)	6.67, dd (13.5, 10.7)	0.04
**19**	6.17, d (13.9)	6.19, d (13.5)	0.02
**20**	0.80, d (7.0)	0.81, d (6.7)	0.01
**21**	1.50, s	1.53, s	0.03
**1′**	4.97, brs	4.99, d (1.9)	0.02
**2′**	3.46, dd (3.0, 1.0)	3.49, m	0.03

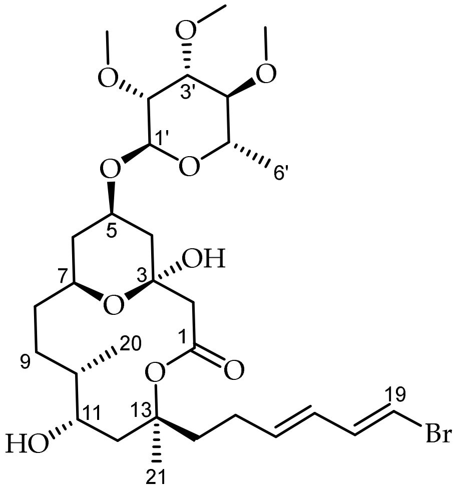

18*E*-lyngbyaloside C (**4**)

**2′-*O*-Me**	3.49, s	3.49, s	0
**3′**	3.42, dd (9.0, 3.0)	3.44, dd (9.4, 3.3)	0.02
**3′-*O*-Me**	3.48, s	3.48, s	0
**4′**	3.08, dd (10.0, 9.0)	3.11, t (9.4)	0.03
**4′-*O*-Me**	3.52, s	3.54, s	0.02
**5′**	3.53, dq (10.0, 6.2)	3.56, m	0.03
**6′**	1.25, d (6.2)	1.27, d (6.2)	0.02

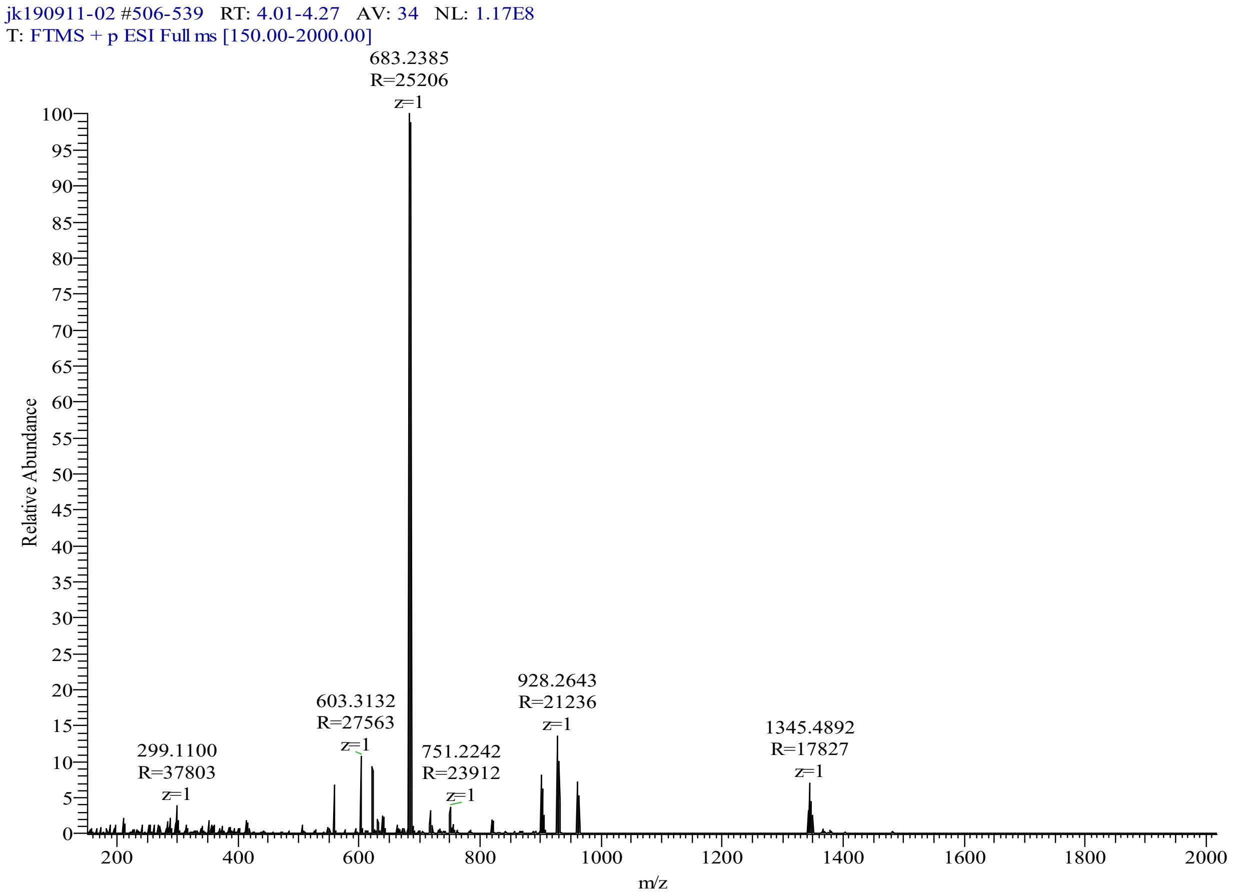

Figure S16. HRESIMS of lyngbyaloside (**5**) *m/z* $[M + Na]^+$ calculated for $C_{31}H_{49}BrO_{10}Na$ 683.2401, found 683.2385 (Δ = 2.3 ppm).

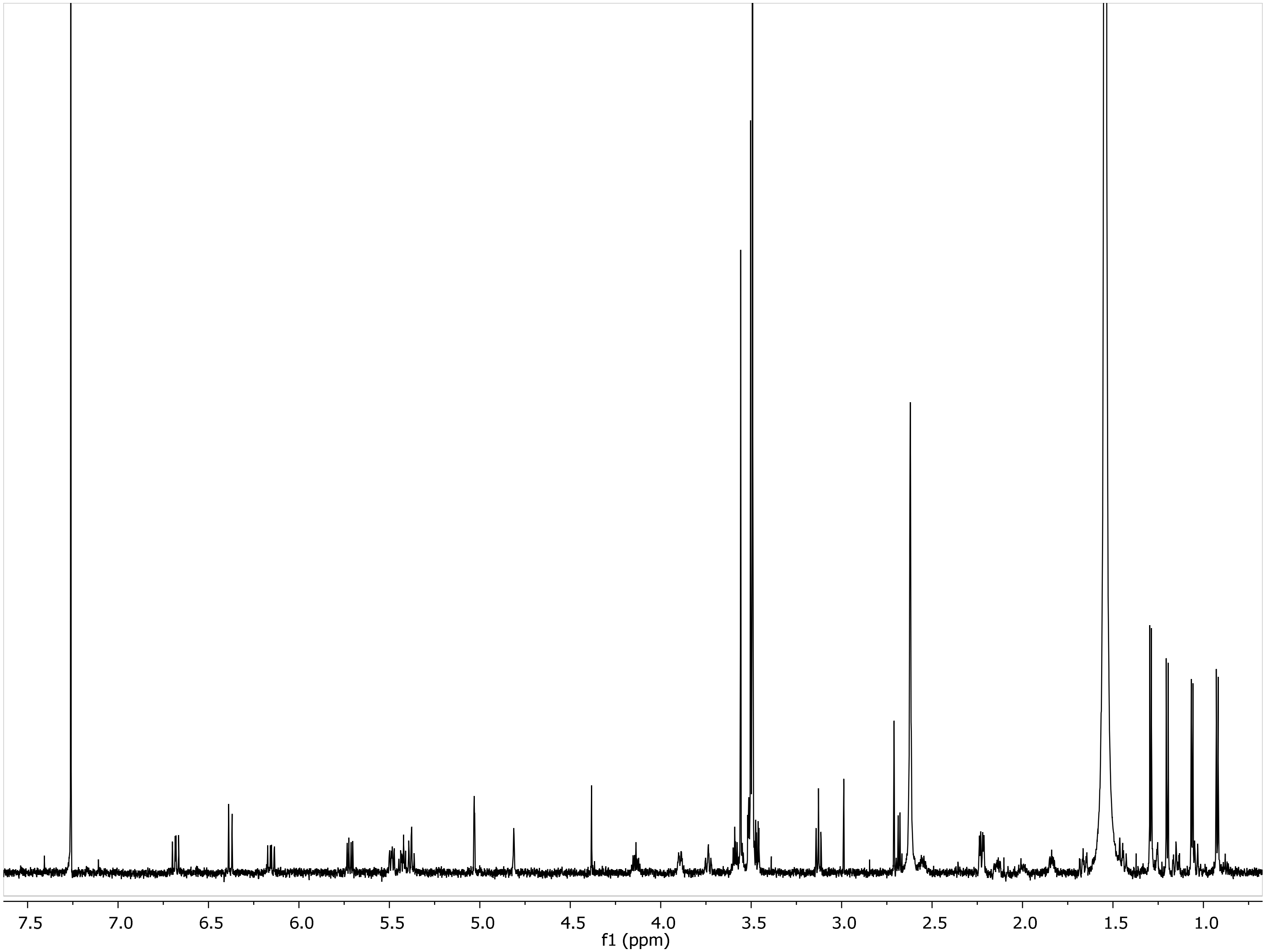

Figure S17. ^{1}H NMR spectrum for lyngbyaloside (**5**) in $CDCl_3$ (700 MHz).

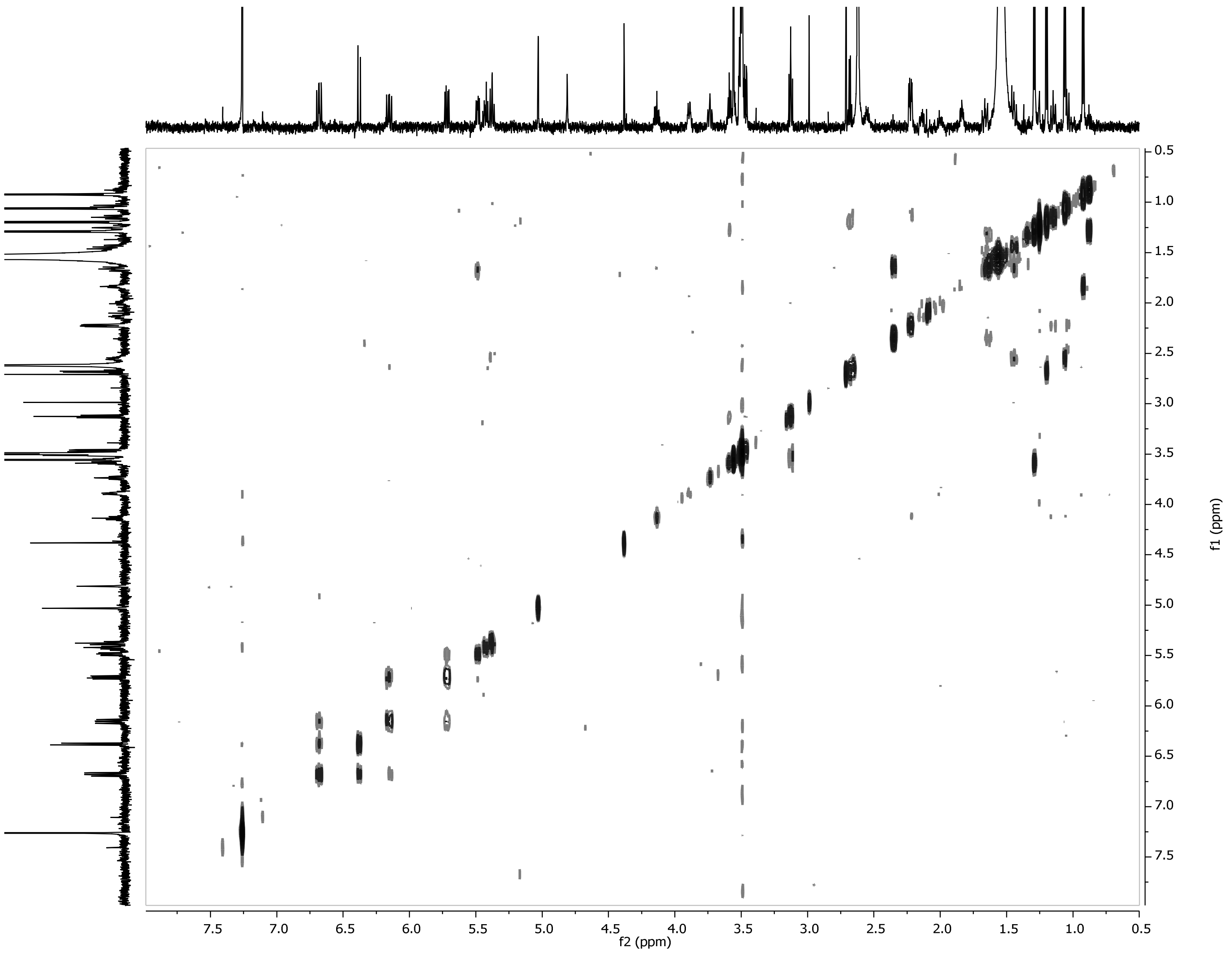

Figure S18. COSY NMR spectrum for lyngbyaloside (**5**) in $CDCl_3$ (700 MHz).

Table S4. Comparison of ^{1}H NMR chemical shifts of lyngbyaloside (5) reported in literature [5] (600 MHz, $CDCl_3$) to experimental values of **6** (700 MHz, $CDCl_3$).

C/H no.	Literature Values	Experimental Values (5)	Δ (ppm)
	δ_H (*J* in Hz)	δ_H (*J* in Hz)	
**1**			
**2**	2.66, q (7.0)	2.68, q (7.0)	0.02
**3**			
**3-OH**	4.79, d (2.5)	4.81, d (1.7)	0.02
**4a**	2.20, dd (12.0, 4.0)	2.23, dd (12.0, 4.5)	0.03
**4b**	1.13, t (12.0)	1.15, t (11.6)	0.02
**5**	4.12, m	4.14, m	0.02
**6a**	2.20, dd (12.0, 4.0)	2.23, dd (12.0, 4.5)	0.03
**6b**	1.02, q (11.5)	1.03, q (11.1)	0.01
**7**	3.73, bt (11.0)	3.73, bt (10.8)	0
**8**	1.82, m	1.84, m	0.02
**9**	3.87, bd (10.6)	3.89, bd (10.6)	0.02
**10a**	2.13, m	2.14, m	0.01
**10b**	1.98, m	1.99, m	0.01
**11**	5.40, dt (10.7, 7.5)	5.43, dt (10.7, 7.7)	0.03
**12**	5.37, t (10.7)	5.38, t (10.3)	0.01
**13**	2.54, m	2.55, m	0.01
**14a**	1.65, ddd (14.0, 10.0, 3.0)	1.66, m	0.01
**14b**	1.42, bdd (14.0, 11.0)	1.44, dd (15.0, 10.7)	0.02
**15**	5.48, bdd (11.0, 6.6)	5.49, dd (11.1, 6.7)	0.01
**16**	5.70, dd (15.3, 6.5)	5.72, dd (15.3, 6.7)	0.02
**17**	6.13, dd (15.3, 10.8)	6.15, dd (15.0, 11.2)	0.02
**18**	6.67, dd (13.6, 10.8)	6.68, dd (13.6, 10.9)	0.01
**19**	6.36, d (13.6)	6.38, d (13.5)	0.02
**20**	1.18, d (7.0)	1.20, d (7.2)	0.02
**21**	0.91, d (7.0)	0.92, d (7.0)	0.01
**22**	1.04, d (6.3)	1.06, d (6.3)	0.02
**1‘**	5.01, d (2.0)	5.03, d (1.7)	0.02
**2‘**	3.52, dd (2.7, 2.0)	3.51, dd (3.3, 1.9)	0.01
**2′-*O*-Me**	3.48, s	3.50, s	0.02
**3‘**	3.44, dd (9.3, 3.3)	3.47, dd (9.3, 3.3)	0.03
**3′-*O*-Me**	3.47, s	3.49, s	0.02
**4‘**	3.11, t (9.3)	3.13, t (9.4)	0.02

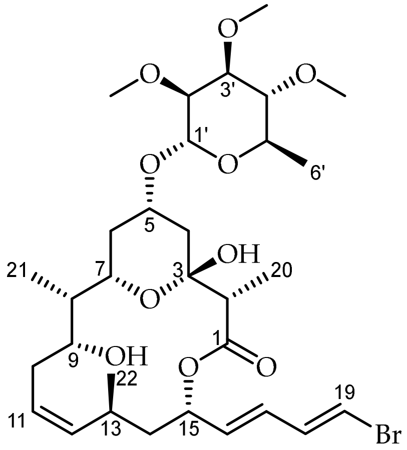

lyngbyaloside (**5**)

**4′-*O*-Me**	3.54, s	3.56, s	0.02
**5′**	3.57, m	3.58, m	0.01
**6′**	1.27, d (7.0)	1.29, d (6.2)	0.02

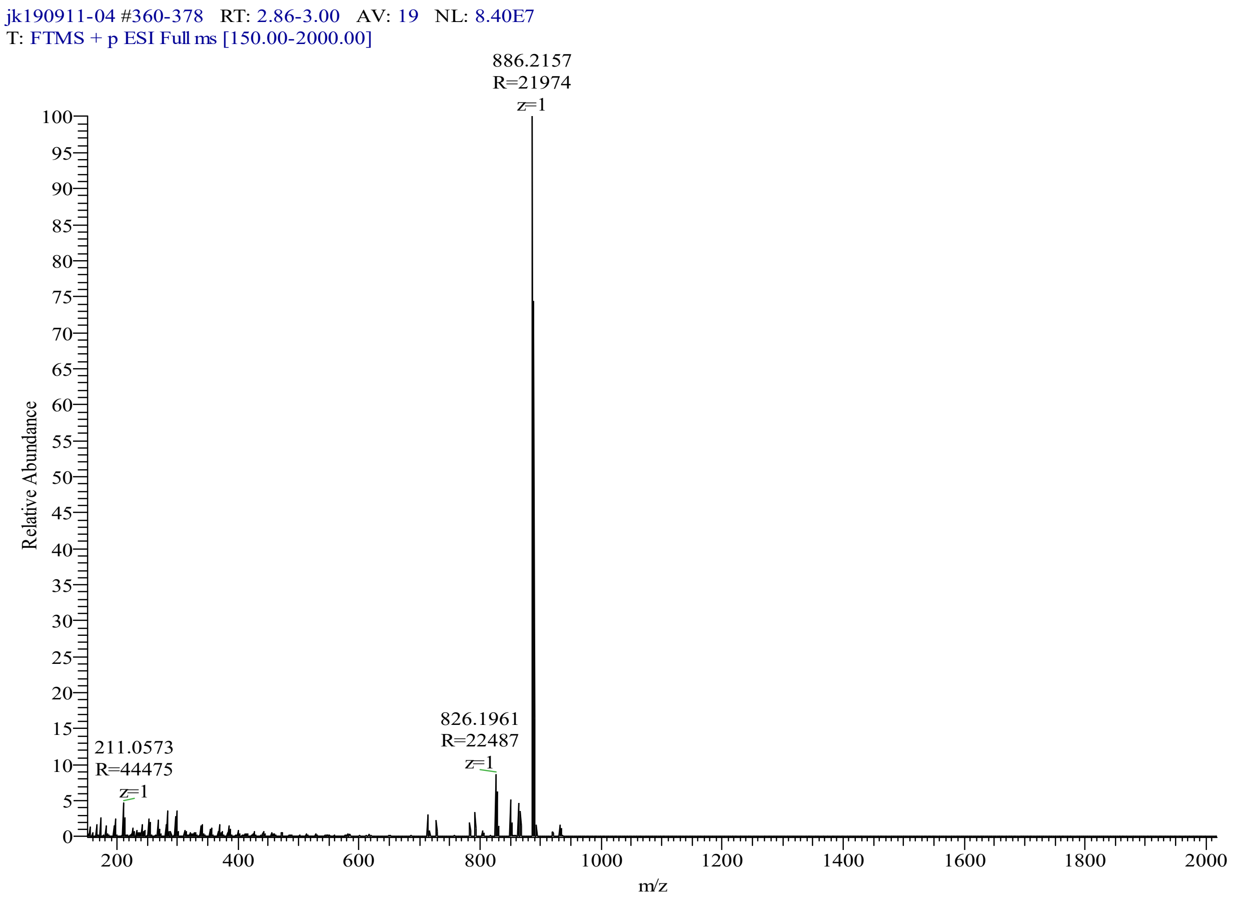

Figure S19. HRESIMS of lyngbyabellin-like 1 (**LYN1**).

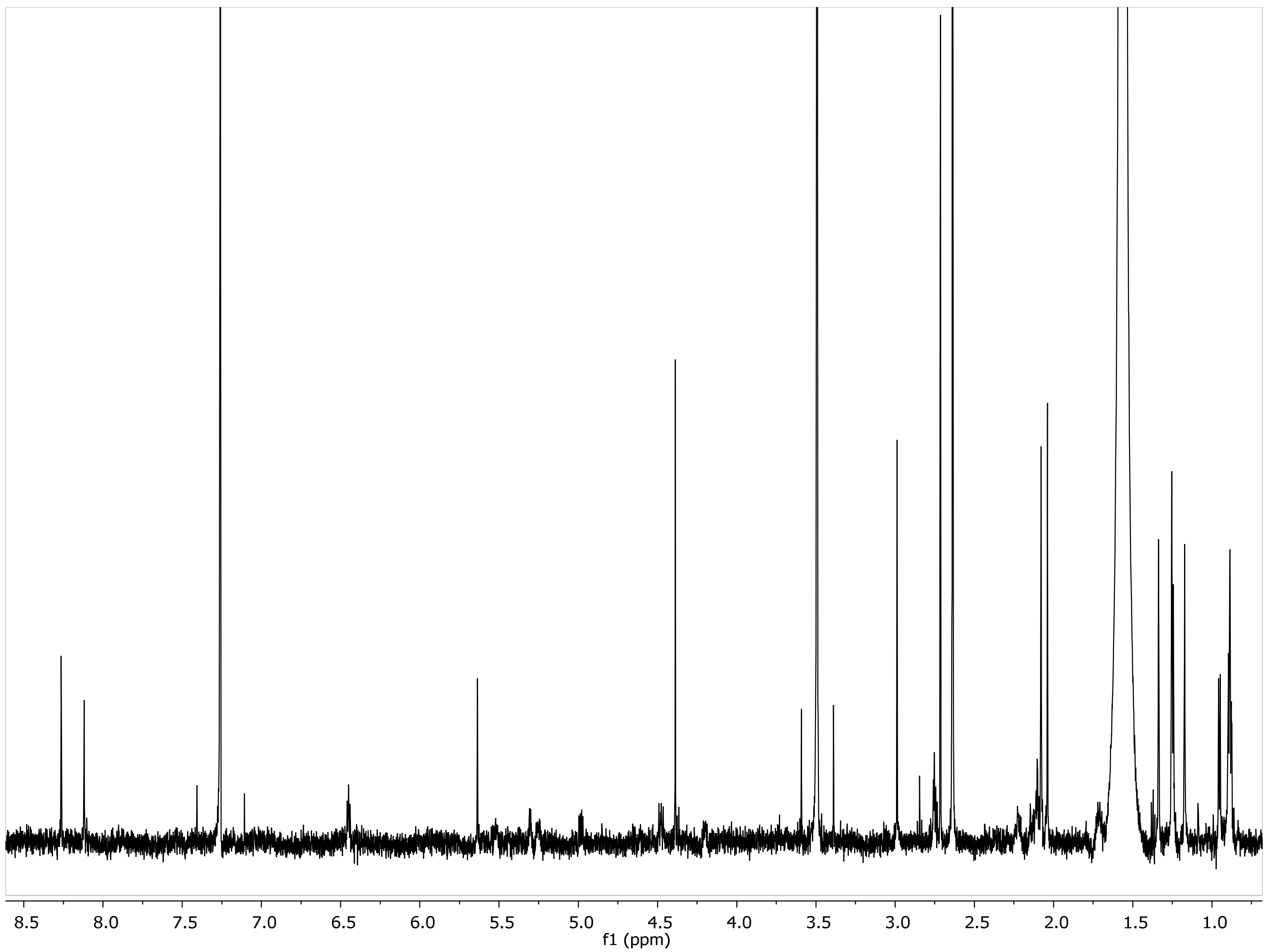

Figure S20. ^{1}H NMR spectrum of lyngbyabellin-like 1 (**LYN1**) in $CDCl_3$ (700 MHz).

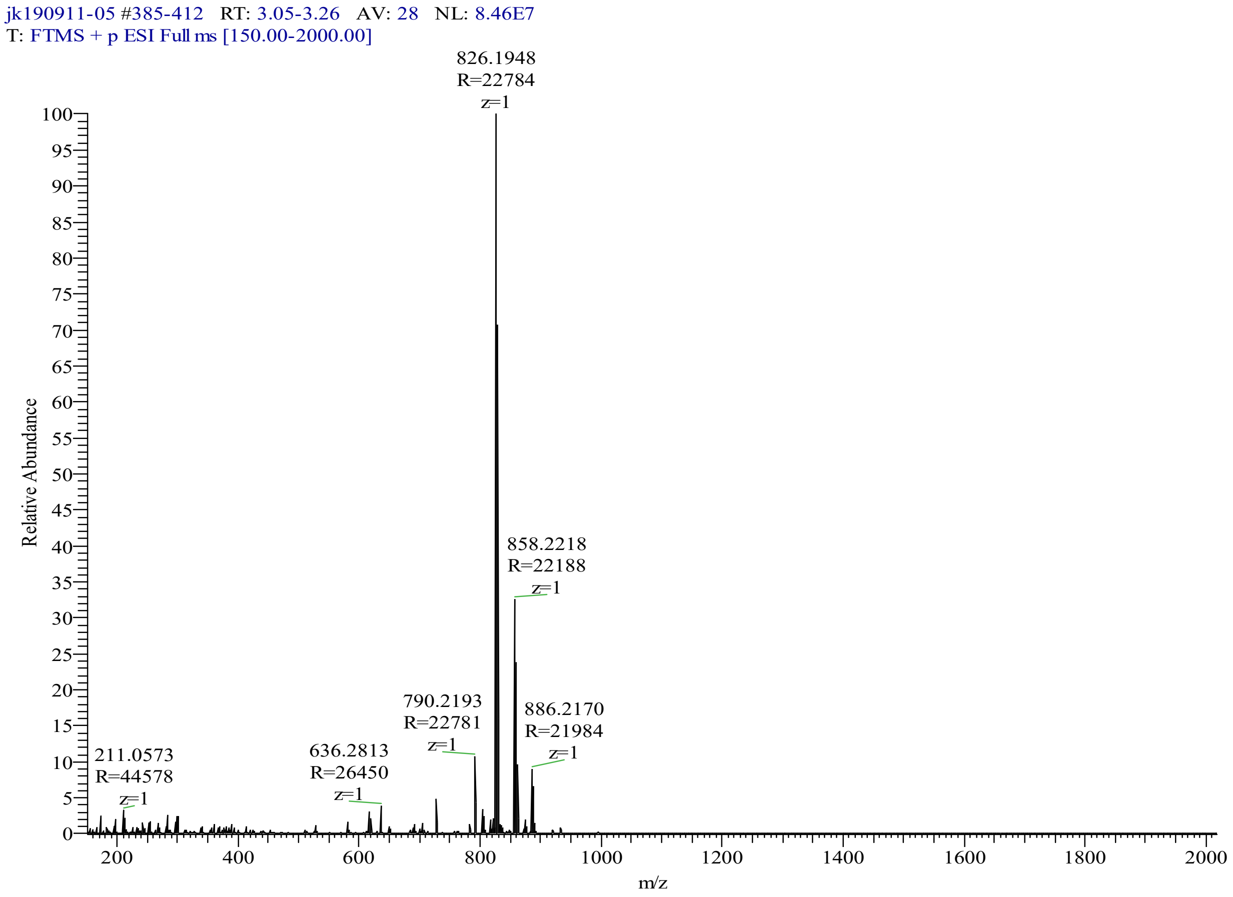

Figure S21. HRESIMS of lyngbyabellin-like 2 (**LYN2**).

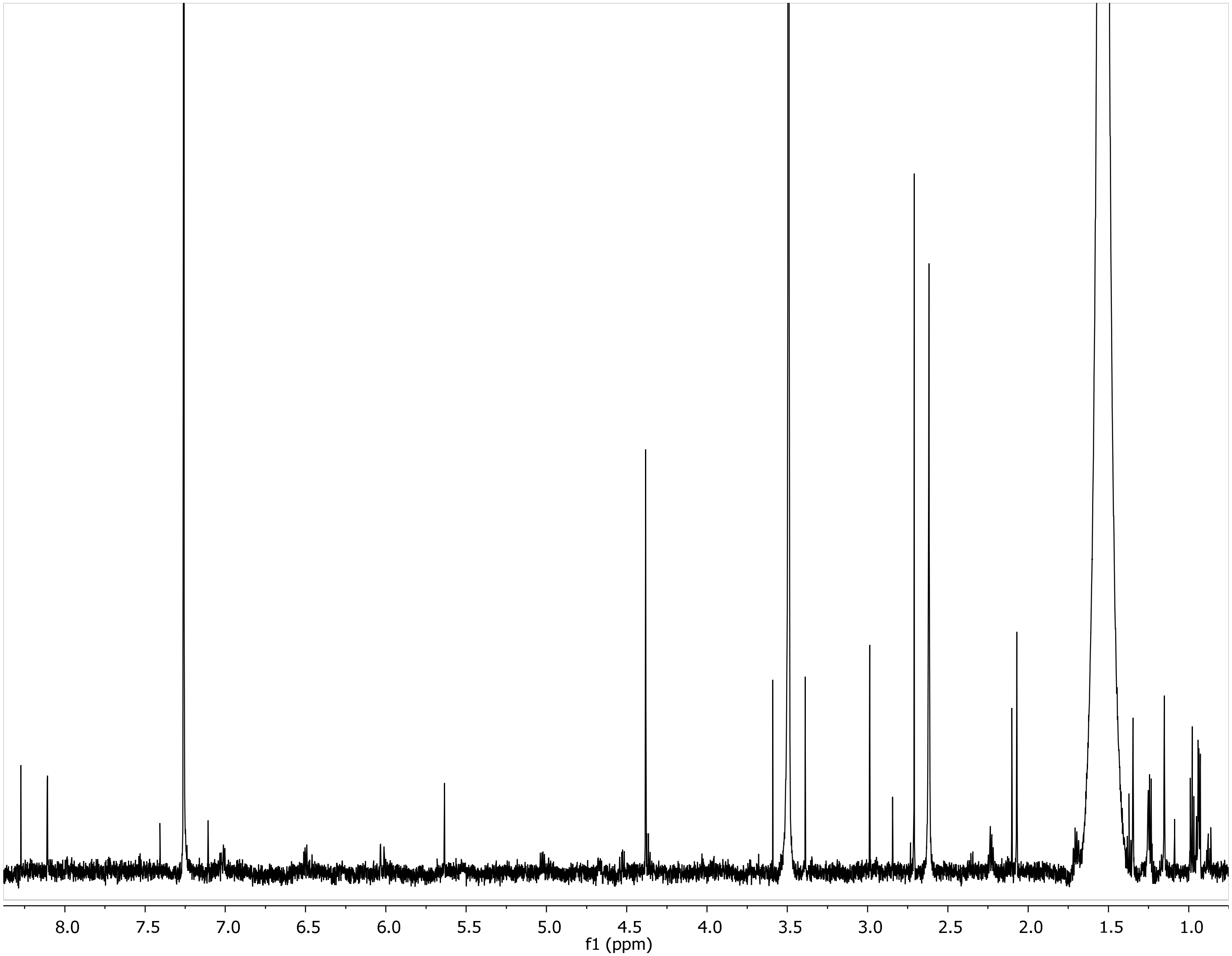

Figure S22. ^{1}H NMR spectrum of lyngbyabellin-like 2 (**LYN2**) in $CDCl_3$ (700 MHz).

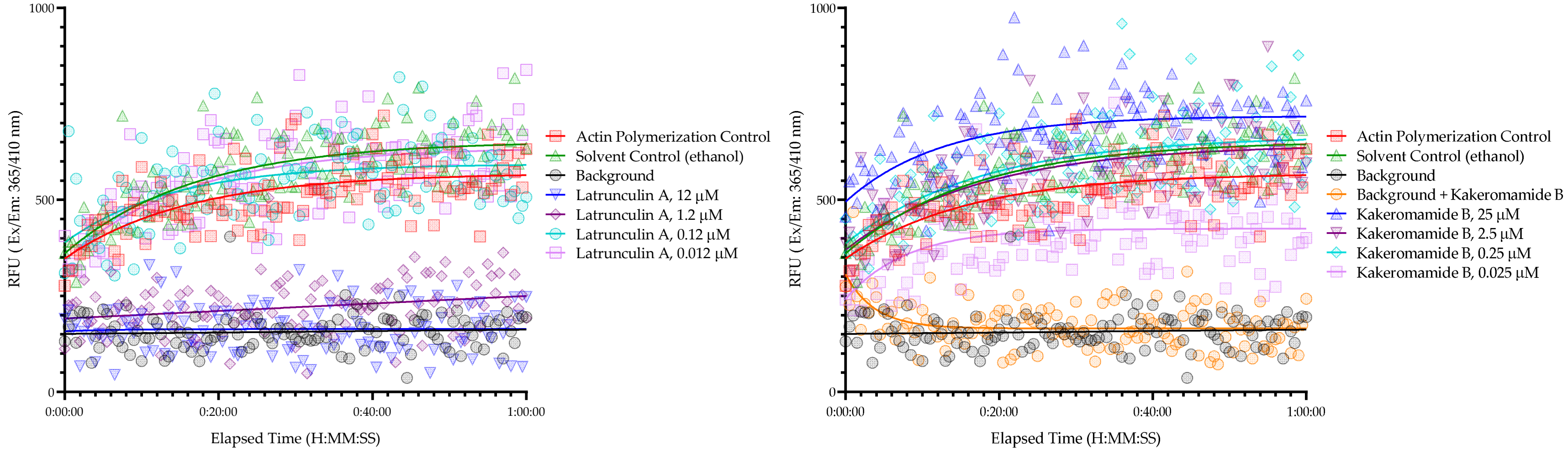

Figure S23. Effect of positive control latrunculin A (left) and kakeromamide B (**1**) (right) on mammalian actin polymerization. Latrunculin A stopped actin polymerization at concentrations of 1.2 µM and above whereas **1** promoted actin polymerization at the highest concentration tested but moderately suppressed polymerization at the lowest concentration tested.

1. Luesch, H.; Williams, P.G.; Yoshida, W.Y.; Moore, R.E.; Paul, V.J. Ulongamides A−F, New β-Amino Acid-Containing Cyclodepsipeptides from Palauan Collections of the Marine Cyanobacterium Lyngbya sp. *Journal of natural products* **2002**, *65*, 996-1000, doi:10.1021/np0200461.
2. Luesch, H.; Yoshida, W.Y.; Moore, R.E.; Paul, V.J.; Mooberry, S.L. Isolation, Structure Determination, and Biological Activity of Lyngbyabellin A from the Marine Cyanobacterium Lyngbya majuscula. *Journal of natural products* **2000**, *63*, 611-615, doi:10.1021/np990543q.
3. Matthew, S.; Salvador, L.A.; Schupp, P.J.; Paul, V.J.; Luesch, H. Cytotoxic Halogenated Macrolides and Modified Peptides from the Apratoxin-Producing Marine Cyanobacterium Lyngbya bouillonii from Guam. *Journal of natural products* **2010**, *73*, 1544-1552, doi:10.1021/np1004032.
4. Fuwa, H.; Yamagata, N.; Okuaki, Y.; Ogata, Y.; Saito, A.; Sasaki, M. Total Synthesis and Complete Stereostructure of a Marine Macrolide Glycoside, (−)-Lyngbyaloside B. *Chemistry – A European Journal* **2016**, *22*, 6815-6829, doi:10.1002/chem.201600341.
5. Klein, D.; Braekman, J.C.; Daloze, D.; Hoffmann, L.; Demoulin, V. Lyngbyaloside, a Novel 2,3,4-Tri-O-methyl-6-deoxy-α-mannopyranoside Macrolide from Lyngbya bouillonii (Cyanobacteria). *Journal of natural products* **1997**, *60*, 1057-1059, doi:10.1021/np9702751.
